# A Semi-Autonomous Telemanipulation Order-Picking Control Based on Estimating Operator Intent for Box-Stacking Storage Environments

**DOI:** 10.3390/s25041217

**Published:** 2025-02-17

**Authors:** Donggyu Min, Hojin Yoon, Donghun Lee

**Affiliations:** Mechanical Engineering Department, Soongsil University, Seoul 06978, Republic of Korea; dg.min0246@gmail.com (D.M.); yhj14531@gmail.com (H.Y.)

**Keywords:** logistics warehouse environment, suction-based telemanipulation order picking, semi-autonomous control method, operator intent estimation

## Abstract

Teleoperation-based order picking in logistics warehouse environments has been advancing steadily. However, the accuracy of such operations varies depending on the type of human–robot interface (HRI) employed. Immersive HRI, which uses a head-mounted display (HMD) and controllers, can significantly reduce task accuracy due to the limited field of view in virtual environments. To address this limitation, this study proposes a semi-autonomous telemanipulation order-picking control method based on operator intent estimation using intersection points between the end-effector and the target logistics plane in box-stacking storage environments. The proposed method consists of two stages. The first stage involves operator intent estimation, which approximates the target logistics plane using objects identified through camera vision and calculates the intersection points by intersecting the end-effector heading vector with the plane. These points are accumulated and modeled as a Gaussian distribution, with the probability density function (PDF) of each target object treated as its likelihood. Bayesian probability filtering is then applied to estimate target probabilities, and predefined conditions are used to switch control between autonomous and manual controllers. Results show that the proposed operator intent estimation method identified the correct target in 74.6% of the task’s duration. The proposed semi-autonomous control method successfully transferred control to the autonomous controller within 32.2% of the total task duration using a combination of three parameters. This approach inferred operator intent based solely on manipulator motion and reduced the fatigue of the operator. This method demonstrates potential for broad application in teleoperation systems, offering high operational efficiency regardless of operator expertise or training level.

## 1. Introduction

The advancement of robotics and teleoperation technologies has significantly enhanced the efficiency and ease of operations of logistics warehouses. Among the diverse tasks in logistics, such as transportation and packaging, Pick-and-Place operations stand out as a primary application of manipulators. Telemanipulation technologies enable operators to handle these complex and demanding tasks more effectively [1,2].

A variety of human–robot interfaces (HRI) are available for telemanipulation in logistics applications. Joystick-based telemanipulation provides relatively simple control, while haptic feedback systems enable fine-grained adjustments of grip forces [3,4]. Virtual reality (VR)- and augmented reality (AR)-based telemanipulation offer an intuitive, 3D understanding of the robot’s movements and its surrounding environment [5,6].

**Figure 1:**
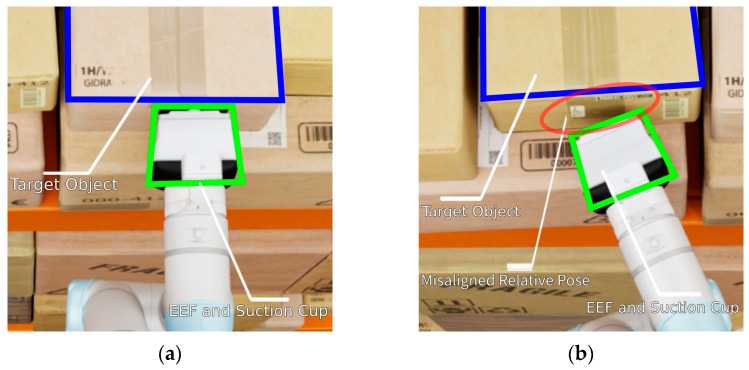
Relative pose between the suction cup and the surface of the target object. (**a**) Successful task execution enabled by a precise relative pose. (**b**) Task failure caused by a misaligned relative pose.

Although these intuitive HRI systems improve the usability of telemanipulation for operators, they often fall short in tasks requiring high precision. Specifically, as shown in Figure 1, the success of picking operations using suction grippers is highly dependent on the relative pose between the suction cup and the surface of the object, as well as the unique characteristics of the object [7,8]. Consequently, careful planning and precise execution of contact locations are critical for effective suction-based grasping. Additionally, in side-picking tasks, a precise relative pose between the suction cup and the surface of the target object becomes even more critical. Unlike down-facing picking tasks, excessive vertical forces applied to the side plane of the target object in these tasks can disrupt the arrangement of other objects. This may exacerbate discrepancies between the virtual and real environments or cause interference with other objects.

In teleoperation environments, operators are generally required to independently plan and execute these contact locations. However, HRI limitations, such as restricted visibility of the workspace, can make it challenging to achieve precise Pick-and-Place operations using telemanipulation systems alone [9]. For instance, camera-based interfaces often fail to provide sufficient depth information for unstructured objects, while VR/AR-based interfaces may face precision challenges in environments with limited visibility [10].

To overcome these challenges, this study proposes a semi-autonomous control method for telemanipulation tasks. The proposed approach predicts the operator’s intent during picking operations, identifies the target object, and employs sensor-based control of the end-effector to perform suction-based picking. This method switches between manual operation by the operator and automated control by the robot, leveraging enhanced precision to improve both the efficiency and accuracy of picking operations in logistics settings [11].

### Related Work

Semi-autonomous control methods for telemanipulation have been extensively studied to improve operational accuracy and reduce the cognitive load on human operators.

- Rubagotti et al. proposed a teleoperation framework integrating Model Predictive Control to enhance task performance in cluttered environments [12]. The study employs a motion capture system to predict the hand posture of a human operator. Based on the data collected from the motion capture system, the hand posture is defined relative to the shoulder joint and used to configure the position and orientation of the robot’s end-effector. The predicted hand posture is then fed into a Model Predictive Control algorithm, enabling real-time replanning of the robot’s motion to avoid obstacles while following movements of the operator.
- Castro et al. proposed a semi-autonomous prosthetic control system that uses a depth sensor mounted on the hand to improve continuous control [13]. This system automatically adjusts the shape, size, and wrist orientation of a prosthetic hand based on environmental conditions. The depth sensor continuously estimates the size and orientation of target objects, allowing for dynamic adaptation until the user is satisfied. This approach enables intuitive interaction by allowing users to understand actions of the system through their movements and adjust their intent accordingly.
- Hauser et al. developed a framework for teleoperation that predicts and recognizes user intent to support autonomous task execution [14]. This framework employs a Dynamic Bayesian Network to analyze user inputs and infer task types using the Freeform Task Inference Engine. The inferred task parameters are passed to a Cooperative Motion Planner, which pre-plans the robot’s movements to follow the predicted trajectory efficiently.
- Menon et al. proposed a shared control system for assistive robots based on user intent prediction and hyperdimensional recall of reactive behavior [15]. Their system leverages Hyperdimensional Computing to analyze electromyography signals and accelerometer data, identifying recent user actions and predicting subsequent actions using probabilistic reasoning. By combining intent recognition with sensor feedback, the system determines the most appropriate operations, reducing the control burden while maintaining autonomy.
- Wang et al. introduced an intent inference method for shared-control teleoperation systems that consider user behavior [16]. Their approach uses Short-Term Path Distance and Short-Term Directionality Deviation to model user behavior, enabling goal prediction and path planning. This method enhances the ability of the robot to accurately understand user intentions and perform tasks aligned with their expectations.
- Jain and Argall proposed a Recursive Bayesian Intent Recognition model for assistive teleoperation systems [17]. This model evaluates joystick input direction and magnitude to calculate the likelihood of achieving the intended target of the user. By estimating transition probabilities in relation to potential goals, the system supports efficient intent recognition and collaborative control in human–robot interactions.

## 2. Materials and Methods

**Figure 2:**
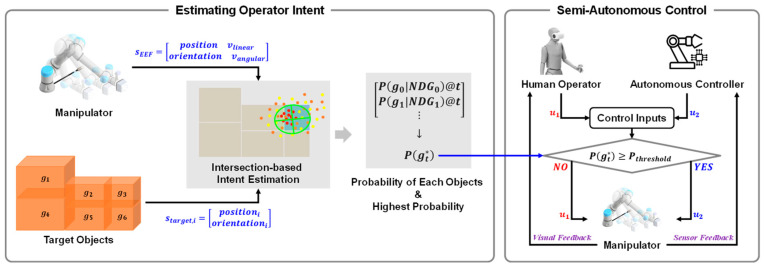
The framework of the proposed semi-autonomous control method. (**Left**) Target object estimation via intersection points and Bayesian probability filter. (**Right**) Determination of control input based on Bayesian posterior probabilities.

In this section, we introduce the components of the proposed system, including the hardware, and then we present the core methods. As shown in Figure 2, the proposed approach consists of two main methods. The first is an operator intent estimation method, which operates at the perception level, and the second is a semi-autonomous control method that transitions based on specific triggers at the control level. A detailed explanation of these two methods is provided in Section 2.2 and Section 2.3.

In summary, the operator intent estimation method utilizes an intersection-based approach, incorporating manipulator motion data and target object position information. This method estimates the likelihood that a given target object is the one the operator intends to pick by analyzing how far the object’s center is from the Gaussian probability distribution derived from the manipulator’s motion, using both the distribution’s center and a covariance matrix.

Furthermore, in the semi-autonomous control method, the probability calculated in the previous step is used to continuously evaluate whether predefined conditions—determined through a parameter study—are met. If the conditions are satisfied, control authority is transferred to the autonomous controller, enabling a transition to fully autonomous control. All controllers in this process ensure reliability through the use of the RTDE Python package provided by Universal Robots.

### 2.1. System Components

This study developed a telemanipulation system where a human operator performs tasks in an immersive environment using an HMD and a controller. An RGB-D camera was installed near the end-effector to enable the robot to identify appropriate contact locations for the suction cup.

The manipulator used in this study is the UR5e, an industrial robot known for its precision and reliability. Excluding the suction cup, it features an 850 mm working radius, a 5 kg payload, and ±0.03 mm repeatability. Its built-in force and torque sensors provide appropriate feedback during the picking process, enabling robust and efficient Pick-and-Place operations.

For the RGB-D camera, Realsense D405 was selected due to its ability to provide high-precision depth data at close ranges. The camera is mounted on the upper side of the end-effector, allowing it to recognize objects in the logistics domain and provide the system with information on contact locations and obstacles. The Realsense D405 has a maximum depth range of 0.5 m, which aligns well with the 850 mm working radius of the manipulator. Additionally, it offers ±0.1 mm depth accuracy, a resolution of 1280 × 800, and a maximum frame rate of 90 FPS, ensuring high performance.

The hardware described above is controlled by a human operator using Meta Quest2 v50 through teleoperation software developed in Unity. This software primarily serves two functions: providing the operator with precise visual feedback in a fully immersive environment and collecting sensor data from the controller.

The process of providing an immersive environment consists of two key steps. The first step involves generating a point cloud map that closely resembles the experimental environment using RTAB-Map [18]. In this study, we used the open-source RTAB-Map package without modifications. The second step is transmitting the generated point cloud map from ROS to the Unity client. During this process, the transmitted point cloud is rendered in the Unity client without any data loss. Through this approach, the operator experiences a fully realized teleoperation environment while seamlessly interacting with the system.

The second function of this software is collecting sensor data from the controller. It gathers gyroscope sensor data from the controller and transmits them to the robot, which then converts the data into appropriate linear velocity commands using manipulator inverse kinematics. Furthermore, when the proposed semi-autonomous control method is activated, the system provides the human operator with haptic feedback signals, intuitively indicating that manual operation is no longer required.

Meta Quest2 was chosen for this study as it provides an immersive experience while simultaneously collecting real-time position and velocity data from the controller. As shown in Table 1, it features a resolution of 1832 × 1920 per eye, supports a refresh rate of 72–90 Hz, and includes four built-in cameras for 6-degree-of-freedom (6 DoF) tracking. This enables precise motion tracking without the need for external sensors. Additionally, the controller is equipped with an accelerometer and a gyroscope, allowing for accurate position and velocity data acquisition.

### 2.2. Estimating Operator Intent

**Figure 3:**
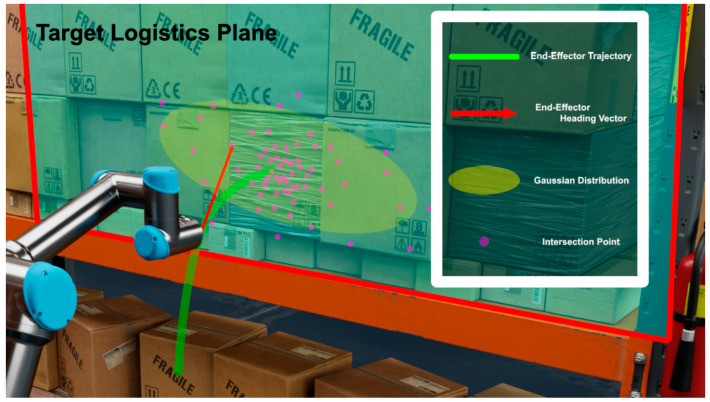
The proposed intersection-based operator intent estimation method for box-stacking storage environments. The green curve represents the trajectory of the end-effector throughout the task; the red arrows indicate the heading vector of the end-effector at specific time steps; the purple dots represent the accumulated intersection points from the start of the task to a given time step; the yellow ellipse is modeled as a Gaussian distribution based on the purple points.

As shown in Figure 3, the proposed intent estimation method of the human operator is designed based on the intersection point between the heading vector of the end-effector and the target logistics plane. Here, the target logistics plane is defined as the approximated plane based on the set of center points of the target objects. This method assumes that as the end-effector moves along the optimal path to pick a specific target, the intersection points generated at a given time *t* exhibit consistent tendencies.

First, the intersection points between the heading vector of the end-effector and the target logistics plane are calculated at each time step *t*. The heading vector of the end-effector is defined as a vector originating from position of the end-effector at time *t* and directed along its linear velocity. The target logistics plane is determined by calculating eigenvectors and eigenvalues from the set of central points on the front-facing surfaces of the target objects. The eigenvector corresponding to the smallest eigenvalue is selected as the normal vector of the target logistics plane. This normal vector is incorporated into the plane equation, and the plane is defined to have a *d* value that best approximates the solution. These intersection points are continuously accumulated from the start to the end of the picking operation, and, at each loop, the set of intersection points is modeled as a Gaussian distribution. Subsequently, the probability density function (PDF) values for all detected target objects are computed based on this Gaussian distribution.

Next, the obtained PDF values are normalized such that its value at the center of the distribution is 1.0. The resulting normalized Gaussian density (NGD) represents the relative distance from the center of the Gaussian distribution, with values increasing as the distance from the distribution center decreases, making it suitable for probability calculations.

**Figure 4:**
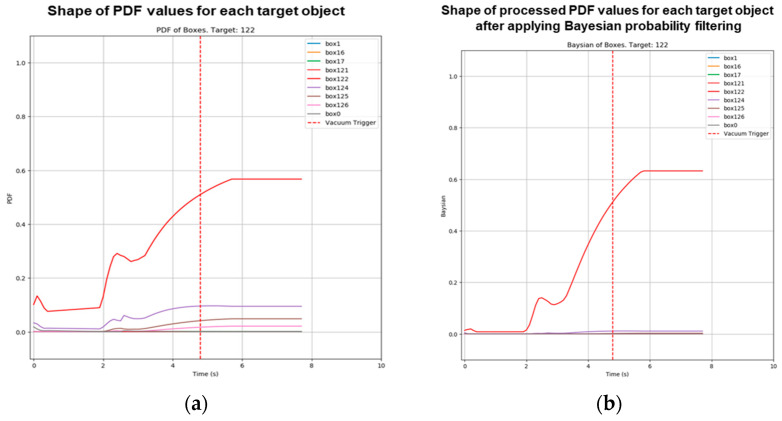
Real-time probability estimation for each target object using the Bayesian probability filter. (**a**) Without the filter, instantaneous probability values are directly reflected. (**b**) With the filter, instantaneous probability values are incorporated into posterior probabilities, resulting in stable and robust probability estimates.

**Figure 5:**
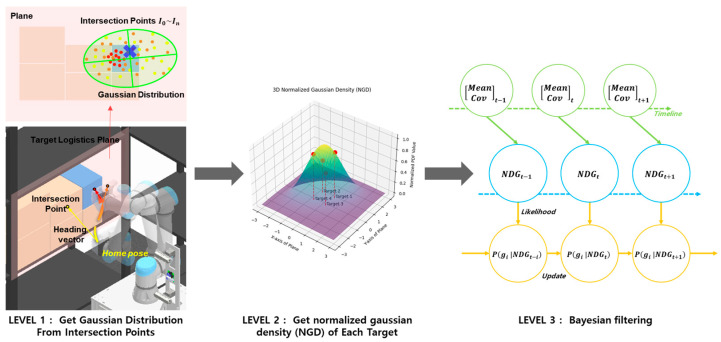
The overall flow of the proposed intersection-based operator intent estimation to obtain the probability of each target object: [LEVEL1] Gaussian distribution modeling; [LEVEL2] normalize the Gaussian distribution; [LEVEL3] application of Bayesian probability filter to independent probabilities.

Intent estimation is conducted using a Bayesian probability-based framework to assign each detected object an independent probability of being the final target object, determined by its NGD value. As shown in Figure 4 and Figure 5, the application of a Bayesian probability filter ensures temporal consistency and mitigates noise in the NGD model, enhancing the stability and reliability of the results. High probabilities observed momentarily are balanced by incorporating prior probabilities, achieving robustness in inference.

Each object is initialized with equal prior probabilities, and the NGD values at each time step are utilized as likelihood inputs within the Bayesian probability filter. These likelihoods are bifurcated into probabilities representing whether an object is the target object or not, enabling the calculation of posterior probabilities [19]. The process is performed recursively in every loop from the start of the picking task, facilitating the cumulative update of inference probabilities for all objects.

By leveraging Bayesian inference, the system seamlessly integrates prior probabilities with new observations to dynamically adjust to uncertainty in operator intent estimation [17]. This recursive approach ensures robust and real-time intent estimation, even in dynamically changing environments [20].

Specifically, each object is initialized with equal prior probabilities, and the NGD values at each time step are used as likelihood inputs in the Bayesian probability filter. These values are bifurcated into the probabilities of being or not being the target object, allowing posterior probabilities to be calculated. This process is repeated in every loop from the start of the picking task, enabling cumulative calculation of inference probabilities for all target objects.

### 2.3. Semi-Autonomous Control Method

The proposed semi-autonomous control method operates based on the geometric information and probability of the most probable target object, as provided by the proposed intersection-based operator intent estimation.

**Figure 6:**
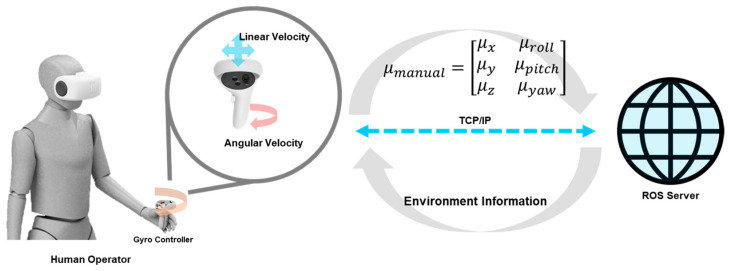
The flow of manual control input: converting gyro sensor values obtained from the HMD controller into appropriate manual control commands and communicating with the ROS server via TCP/IP.

First, as shown in Figure 6, the manual control input from the human operator and the autonomous control input from the autonomous controller are calculated independently. The manual control input is derived from the gyroscope and joystick inputs of the controller in the VR-based HRI, which are processed and converted into linear velocity commands for the end-effector. In contrast, the autonomous control input is determined by the path planner of the end-effector.

Specifically, the optimal path for the target object with the highest probability is generated. This optimal path treats all objects except the target as obstacles that may cause collisions. The final goal pose is determined by selecting the center point of the surface on the target object detected by the RGB-D camera, where the angle between the normal vector of the target logistics plane and the normal vector of the target object surface is minimized. The selected normal vector is defined as the heading vector and used to generate the optimal path.

Path planning algorithms vary depending on the objectives, making the selection of an appropriate method crucial [21]. In this study, the Open Motion Planning Library (OMPL) was employed to generate the optimal path. OMPL is a path planning library that incorporates various sampling-based algorithms, offering advantages like scalability, reusability, rapid computation, and effective obstacle avoidance [22]. This study leveraged OMPL for its fast computation capabilities, enabling real-time updates of the optimal path in every loop.

**Figure 7:**
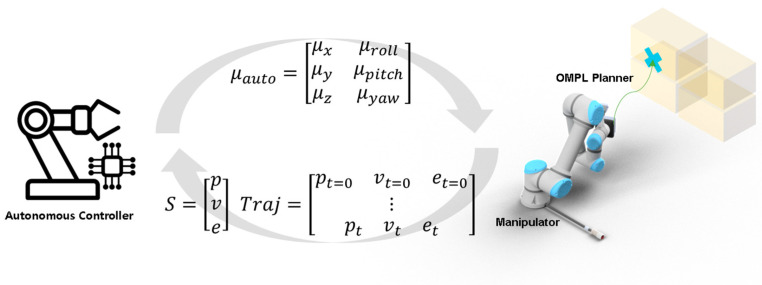
The flow of autonomous control input: generating automated control commands in a closed-loop controller using trajectory data planned by the OMPL planner and state information acquired from the manipulator.

As shown in Figure 7, the path generated by OMPL provides the position, velocity, and effort values required for each joint of the manipulator. During path execution, these joint values are converted into the linear velocity of the end-effector through forward kinematics, ensuring compatibility with the control commands from the VR-based HRI.

The independently calculated linear velocity commands from the VR-based HRI and the autonomous controller are determined based on the Bayesian probability provided by the intersection-based operator intent estimation. This approach infers the intent of the operator from manual inputs and transitions to automated control when sufficient trust is established, aiming to overcome the operational limitations of human operators in the logistics warehouse environment.

Specifically, the Bayesian probability obtained in the preceding stage represents the independent probability of each object being the target object, and three conditions are continuously checked for fulfillment. The first condition is whether the highest probability exceeds the predefined threshold, the second is whether it is significantly higher than the probabilities of other objects, and the last is whether sufficient intersections are accumulated to produce stable data.

The first condition ensures that the highest probability surpasses the threshold, which indicates that the center of the Gaussian distribution continuously aligns with center coordinates of the target object. Consistently high probabilities are required to produce a robust posterior probability through the Bayesian probability filter, making this condition the most critical factor.

The second condition verifies whether the probability difference between the object with the highest likelihood and others is sufficiently large. Because each probability is independently calculated, multiple objects can simultaneously have a high probability of being the target. Thus, even if one object has a high likelihood, the system checks the relative difference to ensure additional stability.

Additionally, in autonomous control mode, unexpected obstacles or interruptions during operation allow the human operator to issue a forced control transfer command to regain control. In the absence of such exceptions, once the task transitions to automation, it remains in automated mode until the task is completed. During this process, the VR-based HRI provides haptic feedback in the form of vibrations to notify the human operator that control has been fully transferred to the automated system. Furthermore, during automated operation, inputs from the preceding intent estimation stage are ignored to address potential exceptions [23].

## 3. Results and Discussion

### 3.1. Experimental Procedure

The experimental design of this study was developed to achieve two primary objectives. First, it evaluates the accuracy of the proposed intent estimation method for the human operator. Second, it examines how quickly and reliably the control authority can be transferred using the proposed method.

Specifically, the manipulator is placed in its home pose, after which the experimenter instructs the human operator, equipped with the VR-based HRI, on which target object to pick. The human operator performs the assigned task, and the experimenter assigns a different target object for each trial. To minimize disturbances caused by the skill level of the operator, the same target object is assigned for at least five repetitions. This process is repeated multiple times with different participants. In addition, during the experiment, the following data are recorded during the experiments.

First, to evaluate the suitability of the proposed intersection-based operator intent estimation, data, such as the center position and the covariance matrix of the Gaussian distribution, the geometric information of all target objects, PDF values, and posterior probabilities derived from Bayesian probability filtering, are collected.

Next, to assess the proposed semi-autonomous control method, raw manual control input, autonomous control input, and combined control input values generated through the intersection-based operator intent estimation are recorded. Additionally, to determine how accurately these control commands are executed, data on the position, velocity, and effort values of each manipulator joint, as well as the linear velocity and angular velocity of the end-effector calculated through forward kinematics, are collected.

### 3.2. Experimental Environment

The experimental environment in this study simulated a logistics warehouse environment, specifically focusing on box-stacking storage. The scenario replicated a mobile manipulator reaching the front of a rack where boxes were stacked. Within the reachable workspace of the manipulator, nine box-shaped objects were placed as task candidates. Each box had a square April Tag measuring 40 mm attached to the center of its front surface. April Tag is a visual marker system designed to ensure stable recognition under various lighting conditions and angles. It allows for precise estimation of the 3D position, orientation, and ID of the tag through cameras, making it well-suited to this experiment [24]. The IDs are unique to each object and randomly assigned without duplication. Moreover, the position of the April Tag, once detected, is updated only when it is deemed valid, ensuring that the final position of the tag and the target is determined based on reliable data. This approach guarantees both the accuracy and continuity of the positional data.

**Figure 8:**
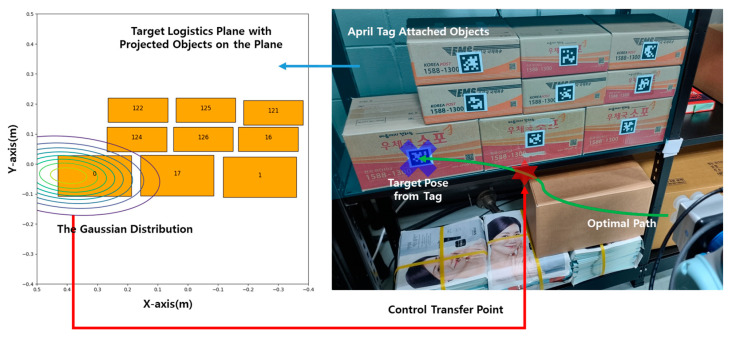
Experimental environment simulating a box-stacking storage logistics warehouse. Right: The actual experimental environment with April-Tag-attached boxes arranged in a 3 × 3 configuration. Target poses are generated based on the tags, and control is transferred according to the defined trigger conditions. Left: Target boxes projected onto the target logistics plane along with the Gaussian distribution. (Note: the *X*-axis is inverted due to the ROS coordinate system). Final posterior probabilities are calculated through Bayesian probability filtering based on this information.

Moreover, the boxes were divided into two size categories: six boxes measuring 220 mm × 90 mm and three boxes measuring 270 mm × 150 mm. This arrangement allowed for the evaluation of performance differences based on box size. The constructed experimental environment is illustrated in Figure 8, and the specific arrangement of each target relative to the base_link frame of the manipulator can be referenced therein.

Additionally, considering the working range of the manipulator, the distance between the mobile manipulator and the target objects falls within a relatively short range, making external interference unlikely during the experiment. Therefore, no additional noise factors were introduced in the experimental setup.

### 3.3. Data Analysis

All experimental data were recorded using the rosbag feature in ROS. Relevant data topics were selected and stored in *.bag format, which were then exported to CSV format. This method ensured that the data timestamps were based on the host system’s time, allowing all data to be saved in a raw state with minimal loss. Because the resulting CSV files were asynchronous logs, the data were merged into 100 ms intervals for ease of processing.

To evaluate the performance of the proposed method, the following topics were recorded and analyzed. These data points were used for performance evaluation and analysis.

- /intention/log/gaussian (geometry_msgs/PoseWithCovarianceStamped): Header, Gaussian distribution center coordinates, and covariance matrix.
- /box_objects/pdf (custom_msgs/BoxObjectMultiArrayWithPDF): Header, geometric information of all vision-recognized target objects, and their PDF values at each time step.
- /control_mode (std_msgs/UInt16): Information about the currently applied mode.
- /desired_box (std_msgs/UInt16): Unique ID of the target object intended by the operator.

Additionally, all data collected in this experiment were recorded in a network-based teleoperation environment, making it essential to verify that network issues did not distort the data. To ensure this, the number of retransmitted packets, latency (RTT, Round Trip Time), and the data transfer rate were measured, with data collection performed using Wireshark software 4.2.10.

### 3.4. Experimental Results

#### 3.4.1. Network Performance Analysis

Before analyzing the experimental data, network packet data were collected during the teleoperation experiments to measure the number of retransmitted packets, the server–client Round Trip Time (RTT), and the data transfer rate per second. The results are shown in Table 2.

The measurements showed an average of one retransmitted packet every 10 s, an average RTT of 5 ms, and an average data transfer rate of 21 kB/s. In general, in real-time communication environments, a network delay of less than 10 ms and a packet loss rate below 1% are known to have minimal impact on system performance. Additionally, considering that high-speed internet offers up to 100 Mbps and gigabit internet supports up to 1 Gbps, the data transmission rate observed in this experiment is well within acceptable limits and does not pose any performance issues.

Comparing these results with established benchmarks, the measured network performance in this experiment is considered highly stable. Therefore, the network environment used in this study is unlikely to introduce data distortion or affect experimental results.

#### 3.4.2. Phase I: Estimating Operator Intent

First, the accuracy of the proposed operator intent estimation method was evaluated by defining a loop where one iteration of the Bayesian probability filter was considered one step and prediction accuracy was measured. Prediction accuracy was calculated as follows:

(1) $$Match\left(t\right)=\left\{\begin{array}{c} 1.0\;if\;g_{t}^{*}=g_{actual}\;AND\;P\left(g_{t}^{*}\right)-P\left(g_{t}^{second-highest}\right)>0.0\\ 0.0\;if\;otherwise. \end{array}\right.$$

(2) $$Accuracy=\frac{\sum_{t=1}^{T}Match\left(t\right)}{T}$$

This configuration was adopted because at the beginning of the task, all Gaussian distributions exhibited excessive uncertainty, resulting in uniformly low and similar prediction probabilities for all objects. Therefore, cases where the highest probability was equal to the second-highest probability were treated as exceptions.

**Figure 9:**
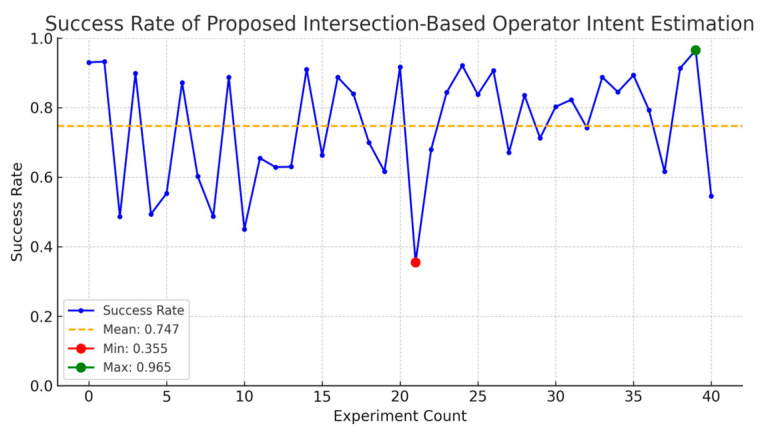
Success rate of proposed intersection-based operator intent estimation.

As shown in Figure 9, the results showed that the average prediction success rate during the task was 74.7%, with a standard deviation of 0.152. Notably, with 50% of the data achieving a success rate of 81.3%, the majority of the dataset demonstrates high success rates. The lower average appears to be influenced by a small number of outliers with low success probabilities. This indicates that the proposed intersection-based operator intent estimation method maintains robust and reliable success rates overall.

Next, during the process of performing suction-based picking with varying targets, the Euclidean distance between the calculated center of the final Gaussian distribution and the actual center of the target object’s front-facing plane was measured. This Euclidean distance will henceforth be referred to as the bias. Considering the dimensions of the box-shaped target objects used in the experiment (width: 220–270 mm; height: 90–150 mm), the average bias was deemed acceptable. However, for IDs 121 and 122 in Table 3, the bias exceeded 100 mm, surpassing the minimum height of some objects, which could pose potential issues. This issue appears to be a fundamental limitation of using the heading vector to generate intersection points. While this approach performs adequately when the center of the target object is relatively close to the origin of the target logistics plane, it was observed to introduce significant offsets when the target object is positioned further away along specific axes.

Additionally, although there were variations in deviation depending on the target box position, the average bias was measured at 77.826 mm. This suggests that the proposed method can effectively determine boxes with a height of up to 80 mm. Furthermore, considering that the suction gripper used in this experiment has four suction cups, which require a minimum contact surface of 60 mm × 60 mm to perform a stable Pick-and-Place operation, this level of bias is deemed sufficiently acceptable.

#### 3.4.3. Phase II: Semi-Autonomous Control

Next, the accuracy of the proposed semi-autonomous control method was evaluated by examining how quickly the predefined thresholds could be exceeded. For this purpose, the semi-autonomous control method was not applied, and the human operator performed the picking task while the results from the Bayesian probability filter were recorded. The following three conditions were applied to these data:

- The highest Bayesian probability exceeds the predefined threshold;
- The difference between the highest and the second-highest Bayesian probabilities exceeds the predefined threshold;
- The total number of intersection points exceeds the predefined threshold.

The first time these three conditions were all satisfied was divided by the total task duration to determine how much of the task had progressed when the conditions were met. Additionally, whether the object with the highest Bayesian probability at that time matched the object the operator intended to pick was recorded.

Multiple thresholds were tested for each of the three conditions. The thresholds ranged from 0.1 to 0.7, 0.1 to 0.5, and 1 to 20, respectively. The results were used to determine the optimal parameters by examining the time at which the conditions were met and whether the identified object matched the intended target.

Specifically, the ranges for three parameters were defined and then sliced into fixed intervals. For each combination of parameters within these ranges, we examined whether the predicted target object matched the predefined target and determined at which stage of the overall task the decision was made.

As shown in Table 4, the resulting dataset was then reorganized by grouping identical parameter values, allowing us to calculate the recognition time ratio and the matching accuracy for each parameter. Although this approach requires significant computational time, it enables a comprehensive analysis of all parameter variations, making it the most accurate method for evaluation.

**Figure 10:**
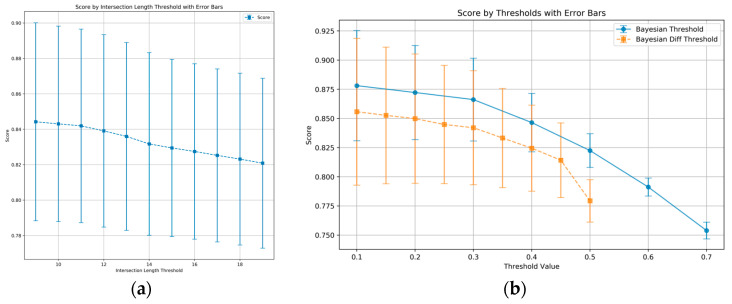
Trend of score values based on predefined parameters. (**a**) Trend of score values with respect to the number of intersection points, showing valid scores recorded only from n ≥ 9. (**b**) Trend of score values based on the highest Bayesian probability threshold (blue line) and the difference between the highest and second-highest Bayesian probabilities (orange line), with lower values for both resulting in better scores.

To evaluate the optimal parameters, the average recognition time and the matching accuracy were converted into scores. This transformation was necessary because the optimal condition in this study requires a low recognition time ratio (RTR) and high matching accuracy (MA), but their scales differ. Therefore, using Equations (3)–(5), we normalized RTR and MA and then applied the weight distribution defined in this study to compute the final score. Its trend is shown in Figure 10.

(3) $${Score}_{RTR}=1.0-\frac{RTR-RTR_{min}\;}{RTR_{max}-RTR_{min}}$$

(4) $${Score}_{MA}=\frac{MA-MA_{min}}{MA_{max}-MA_{min}}$$

(5) $${Score}_{Total}=0.3⨯Score_{RTR}+0.7⨯Score_{MA}$$

Specifically, MA was normalized using a conventional scaling method. However, because RTR should reward faster recognition with a higher score, we applied an inverse normalization by subtracting the normalized value from 1.0, ensuring that it aligns with the same scale as MA. Finally, the two normalized scores were weighted at 0.3 and 0.7, respectively, reconstructing them into a final score ranging between 0.0 and 1.0.

Using these optimal parameters, it was observed that, on average, the target object could be identified at 34% of the total task duration. The average task duration across all tasks was 8.066 s, meaning the target object was identified approximately 2.74 s into the task.

**Figure 11:**
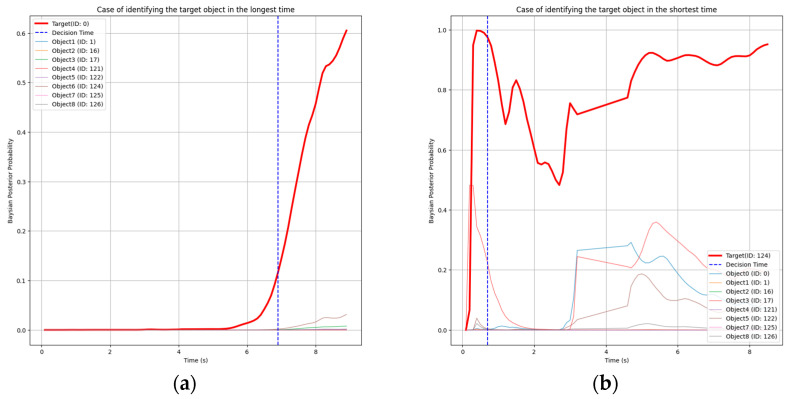
Bayesian probability values (solid lines) for all boxes over time and the decision time (blue solid line) satisfying all conditions when parameters are set to 0.1, 0.1, and 9. (**a**) Case of identifying the target object in the shortest time (8.2% of the total task). (**b**) Case of identifying the target object in the longest time (77.5% of the total task).

In extreme cases, as shown in Figure 11, the target object was identified as early as 8.2% of the task or as late as 77.5%. In the former case, the target box ID recorded a high likelihood from the start, allowing for rapid identification. In the latter case, despite no external interference, the center of the Gaussian distribution was significantly offset from the center of the target object, causing the Bayesian probability filter to exceed the threshold much later.

## 4. Conclusions

This study addresses the limitations of HMD-based immersive HRI in box-stacking storage logistics warehouse environments, including, specifically, the degradation of relative pose accuracy between the end-effector and the target object caused by restricted visibility during the final stages of a task. Existing solutions, such as extending the field of view with additional cameras, compromise intuitiveness, while pure digital-twin-based telemanipulation alone fails to improve the relative pose. To overcome these challenges, we proposed a semi-autonomous control method that estimates operator intent and transitions control to an autonomous controller once sufficient intent inference has been achieved, enabling the determination of the final relative pose.

The proposed method models the set of intersection points between the heading vector of the end-effector and the target logistics plane as a Gaussian distribution. The degree of deviation of each target object from this Gaussian model is calculated and used as the likelihood in a Bayesian probability filter. This approach achieved an average success rate of 74.7% across all tasks, where the proposed inference model correctly identified the object the user intended to pick. Additionally, the average bias between the center of the Gaussian distribution and the April Tag of the intended target object was approximately 77 mm, which is considered sufficiently accurate given the smallest object size used in the experiment (220 mm × 90 mm).

Control transfer was designed to occur when two conditions were simultaneously met: (1) the highest independent probability exceeded a predefined threshold and (2) the highest probability was significantly higher than those of other objects and (3) the number of intersection points was high enough to make stable decisions. This design accounts for the independent nature of the probability model. Experimental results showed that these conditions were satisfied at an average of 34.5% of the task duration. Given an average task duration of 8.066 s, user intent was determined within approximately 2.78 s.

However, some limitations were identified. First, a tendency for the center of the Gaussian distribution to exhibit specific offsets was observed. These offsets were not consistent along a single axis but were distinct and could potentially affect tasks with more densely stacked objects, even though they had minimal impact in the 3 × 3 box-stacking environment used in this study. This is presumably due to the lack of an effective outlier removal process. Therefore, incorporating a method to eliminate such outliers is expected to not only enhance the overall performance of the proposed approach but also reduce variance significantly.

Second, the stability of the control transfer trigger was insufficient. While the recognition time ratio for control transfer was, on average, 34.5%, the standard deviation was 0.256, indicating significant variability. The large difference between the minimum and maximum values further suggests that the trigger was not reliably stable across all tasks. Further studies are required to develop robust and consistent control transfer conditions.

These results stem from the fact that this experiment relies entirely on human operator control. Specifically, cases with Min ARTP occur when the operator’s manipulation follows a nearly linear trajectory and the Gaussian distribution center, derived from the generated intersection points, closely aligns with the center of a specific object. Conversely, cases with abnormally high Max ARTP arise under opposite conditions.

Therefore, future research will focus on refining the Gaussian distribution model to better align its center with the target object’s center or accurately measuring offsets for model correction. Specifically, cases where Max ARTP is abnormally high will be considered outliers, and appropriate filtering methods will be explored to address these anomalies.

Lastly, there are inherent limitations in this study due to its underlying assumptions. In a real warehouse environment, objects of various shapes are often randomly stacked, whereas this study focuses solely on box-shaped objects, with the additional constraint of requiring April Tag for identification. This suggests that in more complex real-world settings, the proposed method may be susceptible to noise factors, potentially affecting its performance.

Furthermore, external disturbances were minimally considered in this study. In actual environments, other robots, human workers, and unexpected incidents introduce significant disturbances. While the working range of the mobile manipulator structurally limits external interference, the absence of explicit consideration of these factors may still result in notable differences between the experimental setup and real-world applications.

Despite these limitations, this study successfully inferred operator intent based solely on manipulator motion, enabling the semi-automation of telemanipulation tasks and reducing operator fatigue.

Additionally, all of the above work was conducted in the GitHub repository at https://github.com/7cmdehdrb/project_semi_remote (accessed on 8 January 2025). The source code and the data, except for the libraries provided by the manufacturers of the RGB-D camera, the suction gripper, the manipulator, and Moveit, were written directly by the authors. These materials are open-source and freely available for anyone to access and use.

## Figures and Tables

**Table 1:** Hardware specifications used in the study and the experiments.

Device	Hardware Information	
UR5e	Working Radius	850 mm
	Payload	5 kg
	Repeatability	±0.03 mm
D405	Maximum Depth Range	0.5 m
	Depth Accuracy	±0.1 mm
	Resolution (RGB)	1280 × 800
	Frame Rate	90 FPS
Meta Quest2	Resolution	1832 × 1920 (each eye)
	Refresh Rate	72–90 Hz
	Tracking DOF ^1^	6 DOF

^1^ Degree of freedom.

**Table 2:** Network performance metrics during the experiment.

Re-Transmission Packet	Round Trip Time	Data Transfer Speed
0.1 packets/sec	5 ms	21.6 kbytes/s

**Table 3:** Average bias and standard deviation between the centroid of the Gaussian distribution and the target object. The target box ID is defined as the unique ID of the April Tag attached to the target box. In addition, the target box IDs are arranged based on the actual placement order of the objects, sorted from left to right and from top to bottom.

Target Box ID	Average Bias (mm)	Standard Deviation
121	100.112	37.987
125	68.55	11.072
122	147.631	29.434
16	44.327	11.611
126	21.236	4.622
124	69.774	25.047
1	56.893	19.343
17	16.777	11.491
0	85.458	23.309
*Total*	77.826	39.093

**Table 4:** The mean, standard deviation, minimum, and maximum of the recognition time ratio for each target box. The target box ID is defined as the unique ID of the April Tag attached to the target box, and the recognition time ratio is defined as the time of successful recognition, normalized to 1.0 using the total task duration. The arrangement of target box IDs is the same as in Table 3.

Target Box ID	ARTR ^1^	STD ^2^	Min ARTR ^3^	Max ARTR ^4^
121	0.364	0.262	0.2	0.667
125	0.612	0.093	0.512	0.697
122	0.494	0.351	0.089	0.705
16	0.372	0.217	0.148	0.582
126	0.113	0.006	0.107	0.119
124	0.132s	0.049	0.094	0.188
1	0.482	0.309	0.127	0.692
17	0.212	0.074	0.134	0.282
0	0.322	0.392	0.093	0.775
**Total**	0.345	0.256	0.089	0.775

^1^ Average recognition time ratio. ^2^ Standard deviation. ^3^ Minimum average recognition time ratio. ^4^ Maximum average recognition time ratio.

## Data Availability

The original data presented in this study are openly available in Zenodo at https://doi.org/10.5281/zenodo.14625181.

## Abbreviations

The following abbreviations are used in this manuscript:

HRI	Human–robot interface
HMD	Head-mounted display
PDF	Probability density function
NGD	Normalized Gaussian density
VR	Virtual reality
AR	Augmented reality
OMPL	Open Motion Planning Library
RTR	Recognition time ratio
MA	Matching accuracy
